# Reducing Risky Alcohol Use *via* Smartphone App Skills Training Among Adult Internet Help-Seekers: A Randomized Pilot Trial

**DOI:** 10.3389/fpsyt.2020.00434

**Published:** 2020-05-27

**Authors:** Anne H. Berman, Olof Molander, Miran Tahir, Philip Törnblom, Mikael Gajecki, Kristina Sinadinovic, Claes Andersson

**Affiliations:** ^1^Center for Psychiatry Research, Department of Clinical Neuroscience, Karolinska Institutet, Stockholm, Sweden; ^2^Stockholm Health Care Services, Stockholm County Council, Stockholm, Sweden; ^3^Department of Psychology, Uppsala University, Uppsala, Sweden; ^4^Division of Psychology, Dept of Clinical Neuroscience, Karolinska Institutet, Stockholm, Sweden; ^5^Department of Criminology, Malmö University, Malmö, Sweden

**Keywords:** alcohol, mHealth, mobile phone apps, pilot trial, randomized controlled trial, risky drinking behavior

## Abstract

Alcohol is one of the leading risk factors for global disease burden and overconsumption leads to a wide variety of negative consequences in everyday life. Digital interventions have shown small positive effects in contributing to reductions in problematic use. Specific research on smartphone apps is sparse and the few studies published indicate effects ranging from negative or null to small or moderate. TeleCoach™, a web-based skills training smartphone app, has shown positive effects in non-treatment-seeking university students with excessive drinking. This pilot trial aimed to evaluate app effects in a sample of internet help-seekers from the general population in Sweden. A total of 89 participants were recruited *via* online advertisement. Following baseline assessment for hazardous use, they were randomized to TeleCoach or a web-based control app offering brief information and advice regarding problematic alcohol use. The primary outcome was number of standard drinks per week; secondary outcomes included drinking quantity and frequency, binge drinking and blood alcohol count measures as well as app user data and comorbidity related to depression, anxiety, and drug use. Analysis of baseline and 6-week follow-up outcomes showed significant within-group effects on alcohol consumption but no significant between-group differences. Effect sizes for the within-group changes in the primary outcome over time were significant [*F*(1, 55)=43.98; p < 0.001], with a Cohen's *d* of 1.37 for the intervention group and 0.92 for the control group. This difference in effect sizes indicated that continuation of the study as a large randomized, controlled trial with up to 1,000 participants could be worthwhile.

**Clinical Trial Registration:**

www.ClinicalTrials.gov, identifier NCT03696888.

## Introduction

Alcohol is one of the most commonly used and socially accepted psychoactive substances worldwide, with overconsumption leading to significant impacts not only on individual levels but also on societal levels. Indeed, World Health Organization (WHO) reports consistently show that alcohol is ranked among the top leading risk factors for the global disease burden associated with premature death and disease. In 2016, 3 million deaths worldwide were attributed to hazardous or harmful use of alcohol. A total of 132.6 million disability-adjusted life years (DALYs), were attributable to alcohol, an equivalent of 5.1% of all DALYs in the year of 2016. Injuries, cardiovascular diseases and digestive diseases resulting from harmful alcohol consumption were among the top leading factors behind alcohol-related deaths in 2016 (1). In addition to the health-related consequences of increased alcohol consumption, it also leads to major financial consequences that burden society, including an increased need of health care, increased criminality and a loss of productivity. Implementing interventions on a societal level to lower these economic burdens could potentially save hundreds of millions of dollars per year (2).

Overconsumption of alcohol can also lead to diagnosis of mild to moderate alcohol use disorder [AUD; (3)], with prevalence estimated at 8.8% in Europe (1). AUD can involve binge drinking, defined as alcohol consumption exceeding five standard units per occasion. Individuals with AUD show high comorbidity with other psychiatric disorders, particularly mood disorders, in the general population, as shown recently in Danish (4) and South Korean (5) cohorts, with the latter showing specific comorbidity with anxiety and depression disorders as well as bipolar disorder and higher risk of suicide. Although AUD is one of the most globally prevalent mental disorders, there is a large disparity between the number of people with AUD and the number actually receiving adequate treatment. This disparity, termed the treatment gap, has been estimated to be as high as 92.4% in Europe (6). Barriers to seeking help have been shown to include stigmatization, absence of trust or belief for the effectiveness of treatment and denial are among the top barriers to help seeking (7).

In Sweden, AUD prevalence is 11% for men and women combined (14.7% for men and 7.3% for women), higher than the European Region average of 8.8% (1), and the percentage of Swedish adult hazardous drinkers with high-risk consumption or at least one episode of binge drinking over the last 30 days has been identified at 31% (8). Barriers to treatment among Swedish respondents with AUD include feelings of shame and stigma, and preferred treatment should feature low-threshold access, allow for high personal autonomy, and not interfere with everyday life activities (9). Digital interventions conform to these preferences, and international research from several countries has shown small but promising overall effects in reducing problematic alcohol use (10), with small effects found for self-guided Cognitive-Behavior Therapy (CBT) programs and small to large effects for guided programs (11). An accumulating body of research in Sweden has shown the promising effects of such interventions for reducing hazardous and harmful use of alcohol (12–14) as well as diagnosed AUD among Internet help-seekers (15). Research has also indicated increased general well-being up to 1 year after participation in digital interventions for reducing alcohol use (16).

The delivery of digital interventions for general mental health issues *via* increasingly ubiquitous smartphone apps is gaining significant ground, but significant challenges exist regarding the evidence for their effectiveness (17). Indeed, of the great number of alcohol-related apps available on the market, an early review showed that very few aimed to reduce drinking (18). Among the apps that do address reduction of problematic drinking, effectiveness research is scarce and a recent systematic review identified six apps evaluated in five different studies, with only two showing positive results in reducing drinking (19). The first, A-CHESS, targets individuals during and after residential treatment for AUD, and has led to fewer days with risk-level drinking compared to controls who received treatment as usual without access to the app, up to 4 months after leaving residential treatment; users accessed the app on 41% of days and 72% pressed a panic button at least once (20). The second, TeleCoach, has been evaluated among non-treatment seeking university students with excessive drinking beyond the limit of nine/fourteen standard units per week indicated by public health recommendations; access to TeleCoach has led to lower proportions of excessive drinking and reduced quantity and frequency after six and 12 weeks compared to waitlist controls; no user data were available (21). In a secondary analysis, the TeleCoach app has also shown positive effects among a latent class of student drinkers with frequent-heavy patterns of drinking, who differed from students with hazardous drinking who reported drinking on only 1 day a week (22). Two additional smartphone apps, not included in the abovementioned review (19), have targeted adult help-seekers. One, targeting internet help-seekers with harmful levels of alcohol use (15 or more drinks/week), did not find any effects over 6 months, although some benefit appeared to occur among those who actually downloaded the app (23). A fourth smartphone app, Drink Less, evaluated among adult help-seekers with at least hazardous alcohol use who all downloaded the app, showed declines in alcohol use over time among all participants, where self-monitoring and feedback combined led to use of more app sessions (24); secondary analysis using Bayes factors showed weak evidence for an interactive combined effect of four components (normative feedback, cognitive bias retraining, self-monitoring and feedback, and action planning) yielding lower alcohol consumption (25).

Research studies on smartphone apps for reducing problematic alcohol use among adults motivated to seek help *via* the internet are clearly scarce and have not, as yet, shown any clear positive results. Prior studies of the TeleCoach app were conducted among university students directly targeted *via* e-mail (21, 22). Uncertainty regarding the feasibility of recruitment among internet help-seekers, as well as the potential acceptability and usage levels of the two newly designed apps to be compared, motivated the analysis of initial recruitment and 6-week follow-up data as a randomized pilot trial with feasibility assessment features (26). The pilot trial was thus an analysis of the first wave of data in the already planned full, randomized, controlled trial (RCT). The primary research question concerned comparison of the two apps in terms of indications of effects on past week drinking, with secondary outcomes measuring alcohol consumption in terms of drinking quantity and frequency, binge drinking and blood alcohol count measures, as well as self-efficacy with regard to abstaining from drinking. Given prior limitations in reported assessment of user data for the TeleCoach app [see (21)], a second question concerned overall app use and use of app components, where the intervention and control apps were compared. In view of the known high levels of comorbidity among individuals with problematic alcohol use, a third question concerned description of participant characteristics, including assessment of depression, anxiety and drug use, as well as management of depression- and drug-related comorbidity among potential participants. If no significant changes are made in study design, the pilot interim data will be included in the main trial.

## Materials and Methods

### Sample

Participants were recruited *via* Google AdWords, with a link to an online screening survey. The ads appeared among the top results for Google searches including key terms in Swedish such as “alcohol problems”, “alcohol help”, and “alcoholism”. In the screening survey, participants received information about the possibility of winning an iPad lottery if they completed all follow-ups, at 6, 12, and 26 weeks. The sample size was based on the numbers recruited within a total data collection period of 18 weeks including 6-week follow-up.

The inclusion criteria were: age over 18, and hazardous alcohol use, defined as a score of ≥6 (women) or ≥8 (men) on the Alcohol Use Disorders Identification Test [AUDIT; (27)], with differing criteria by gender in accordance with evidence-based praxis in Sweden (28). Exclusion criteria were: severe depression, as defined by scores of >30 on the Montgomery Åsberg Depression Rating Scale (MADRS-S) and/or suicidal ideation as defined by a score of >5 on question 9 of the MADRS-S (29), and/or scores of ≥8 on the Drug Use Disorders Identification Test [DUDIT; (30, 31)]. Participants who met exclusion criteria were contacted by authors AHB or OM (both licensed psychologists) for a brief clinical interview by telephone, and offered referral elsewhere if appropriate. Participants who did not respond were excluded from the study. However, those who were interviewed and wanted to participate in the study, in spite of fulfilling exclusion criteria for the study, were allowed to participate, on condition that follow-up interviews were planned. As described above, exclusion criteria concerned high levels of self-reported depression, suicidal ideation or moderate drug use. The reasoning behind inclusion for these individuals was that interviewed participants had explained the background to their self-reports; e.g., that despite depressive symptoms they were still functioning at work and in family and social roles, that their suicidal ideation was variable and that they had no concrete plans of committing suicide, and/or that their drug use was highly sporadic or that they had misunderstood the drug questionnaire. Eleven participants who met exclusion critiera did not respond to contact attempts; five additional participants who had met exclusion criteria at baseline were included in the study after the telephone interview.

It should be noted that inclusion and exclusion criteria were amended after 2 weeks of recruitment, to conform to the above description. According to the initial study protocol, the inclusion criterion of risky alcohol consumption was defined as alcohol consumption over public health guidelines in Sweden, stipulating standard unit consumption (12 grams of alcohol) of >9 (women) or >14 (men) (32). An additional exclusion criterion required participants to meet ≥6 AUD symptoms according to the DSM-5. In combination, these criteria led to very few individuals meeting the criterion for risky alcohol consumption without also meeting the DSM-5 criterion for AUD, resulting in exclusion of most potential participants. These criteria were therefore amended to the ones described above, and approved by the Swedish Ethical Review Board 4 weeks after recruitment began (2018/2569-32). Three individuals were excluded due to meeting the AUD-criterion before the amendment was approved.

### Procedure

Participants completed the screening survey after giving their informed consent. Those who did so, and met inclusion criteria (manual assessment), were referred to an online baseline assessment survey including demographic information, gender and weight data for calculation of estimated blood alcohol concentration (eBAC), and further questions on alcohol consumption, readiness to change, abstinence self-efficacy, cravings, and anxiety. After completion of the baseline survey, participants were randomized to receive either the intervention or control app, which were both web-based and accessed *via* any web browser on either smartphone or computer. Participants were randomized at a 1:1 ratio through 50 x 20 block randomization (33). Study authors were blinded to the randomization process. Participants were informed that they could be randomized to one of two different apps containing supportive measures aimed to decrease risky alcohol consumption. Six weeks following inclusion, a follow-up online survey was sent out. E-mail reminders were sent to participants beginning 1 week after non-complation of the 6-week survey. The initial procedure stipulated three reminders, but in view of the pilot nature of the initial data collection and with the aim of reducing attrition, additional weekly reminders were sent until the pilot trial cut-off date to participants who did not respond. The data reported for this trial therefore include all participants who responded by the cut-off date.

**Figure 1:**
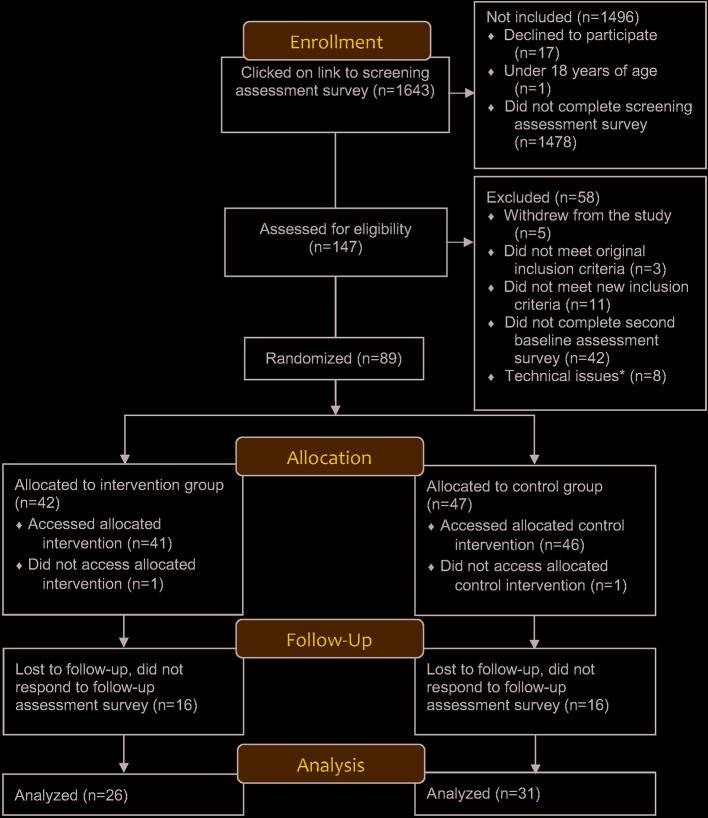
Participant flow diagram. Completed baseline assessment survey but were not randomizeddue to entering incorrect login codes or not activating codes in time (34).

Participant recruitment began on December 4^th^, 2018 and continued until February 15^th^, 2019. The final distribution of the 6-week follow up survey occurred on March 29, 2019. All data collected by April 9, 2019 were included in this pilot study. Of the 1,643 persons who clicked on the study link to the study, 9% completed the screening survey and were assessed for eligibility (n=147). Of these, 60.5% completed the baseline assessment survey and were randomized (n=89), with 64% of these responding to the follow-up survey (n=57; 26 in the intervention group and 31 in the control group). Two participants were lost at randomization because they did not access their allocated interventions. Missing data occurred at baseline because aside from those who withdrew, did not meet original or new inclusion criteria or encountering technical issues, 42 participants who were found eligible for the study at the initial screening elected not to continue to the baseline assessment survey. At 6-week follow-up, missing data occurred because 16 participants in each group simply did not respond to our invitations to participate in the follow-up survey. **Figure 1** displays the participant flow throughout the pilot trial.

### Measures

The initial screening survey included measures of risky alcohol consumption, alcohol use disorder, depression and drug use disorders. Due to the changes in inclusion and exclusion criteria described above, in this pilot study the new inclusion criterion measure of the AUDIT was part of the baseline assessment survey. The baseline assessment survey also contained measures of participant readiness to change, alcohol cravings, symptoms of generalized anxiety disorder and self-efficacy regarding the confidence to abstain from alcohol.

#### Initial Screening Survey

##### Timeline Followback (TLFB)

The TLFB requires respondents to report their alcohol consumption in number of standard drinks for each day for a specified period of time up to 1 year. The questionnaire is accompanied by an instruction in which respondents are presented with examples of what equals a standard glass, where they begin with reporting the day before and go backwards 1 day at a time. The TLFB has become one of the most used instruments for measuring substance consumption and its psychometric properties have been evaluated with positive results both in its standard interview administration form and in web-based administration (35–37). The version used in this study covers the past 7 days (38).

##### Screening for Alcohol Use Disorders (AUD)

AUD assessment was carried out *via* self-report, using 11 questions based on a validated, authorized Swedish translation of a US self-report version of the diagnostic criteria for alcohol use disorder according to the DSM-5 (39, 40).

##### Montgomery-Åsberg Depression Rating Scale (MADRS-S)

Depression symptoms were measured *via* the Montgomery-Åsberg Depression Rating Scale (MADRS-S), a self-rated depression scale where participants rate nine items regarding their current state of mind on a Likert scale. Each item is scored 0–6, with scores over 30 indicating risk of severe depression. Item 9 concerns suicidal ideation and scores over 5 indicate suicide risk. The psychometric properties of MADRS-S have proven to be very good (29).

##### The Drug Use Disorders Identification Test (DUDIT)

The 11-item DUDIT (28) was used to measure participants' problematic drug use and includes a list of common illicit drugs in different categories. Items 1–9 are scored 0–4 and items 10–11 are scored 0–2–4. Reliability and validity have been evaluated and replicated in multiple studies (41).

#### Baseline Assessment Survey

##### Alcohol Use Disorders Identification Test (AUDIT)

The 10-item AUDIT was originally developed by the WHO (26) and is a screening test for identifying hazardous drinking, based on alcohol consumption and alcohol-related problems related. Items are scored 0–4 for the first eight items and 0, 2, and 4 for the last two. The psychometric properties of the instrument have proven suitable for primary care use (28).

##### The Daily Drinking Questionnaire (DDQ)

In the DDQ, participants are asked to register their alcohol consumption for a typical week during the past month. In addition to registering the amount of standard drinks consumed for every day of the typical week, the time interval in which these drinks were consumed is also reported in the form of hours. Participants are also asked about the occasion in which they consumed the most alcohol during the past month and report the number of drinks and the time interval for that day. Evaluations of the psychometric properties of DDQ have shown positive results in regard to the instrument's reliability and validity (42). The DDQ yields measures of quantity, frequency, binge occasions, average estimated blood alcohol concentration (eBAC) and peak eBAC. Quantity is based on the number of standard glasses consumed in a typical week of the past month, frequency is calculated from the number of days participants consumed alcohol in the typical week, and binge occasions build on the number of reported days participants engaged in binge drinking, defined as four or more standard drinks for women and five or more for men. Average eBAC is calculated based on the Widmark formula according to the procedure described in (43) for the 7-day typical week, and peak eBAC is calculated from the event with the highest level of alcohol consumption in the past 30 days. The DDQ makes it possible to make more exact and varied calculations regarding several different parameters of alcohol consumption in a typical week in the past month, in comparison to the 7-day version of the TLFB, which describes the number of drinks in the past 7 days.

##### Readiness Ruler (RR)

RR consists of a Visual Analogue Scale (VAS) that measures how ready participants are to change their behavior, in this case drinking habits. Participants rated readiness to change on a scale of 0–10 ranging from “I am not ready to change my drinking habits” (0) to “I am very much ready to change my drinking habits” (10). RR is often used both clinically to facilitate behavior change in connection with Motivational Interviewing [MI; (44)] and in research to assess participant readiness for change (45).

##### The Penn Alcohol Craving Scale (PACS)

The 5-item PACS is a self-assessed craving scale concerning thoughts about drinking, especially the intensity and frequency of the cravings, where each item is rated 0–6. The psychometric properties of PACS have proven both valid and reliable for predicting individuals at risk for relapse into problematic drinking (46).

##### Generalized Anxiety Disorder (GAD-7)

The GAD-7 is a brief, 7-item self-report screening instrument for Generalized Anxiety Disorder (GAD), developed based on DSM-IV diagnostic criteria (47). Subjects are asked to rate how often they have experienced the seven GAD symptoms during the past 14 days on a scale of 0–3. Studies evaluating GAD-7 psychometric properties have provided strong support for its reliability and validity (48, 49).

##### The Alcohol Abstinence Self-Efficacy Scale (AASE)

The AASE measures self-efficacy regarding the self-perceived confidence in abstaining from drinking in 12 risk situations, divided into four subscales with three risk situation questions each. The subscales are termed negative affect, social positive, physical and other concerns, and withdrawal and urges. The scale has shown high levels of reliability and validity in previous studies including psychometric evaluation (50).

### Outcome Measures

The primary outcome measure of alcohol consumption was the change in total number of standard drinks consumed for each of the 7 days in the preceding week, using the TLFB. Secondary outcome measures of alcohol consumption were based on the DDQ. Six-week follow-up also included the AASE. Twelve-week follow-up (not reported here) will include the same measures as in the 6-week follow-up. The 26-week follow-up, (not reported) will include all measures from the initial and baseline screenings as well as questions on access to other treatment forms during the study (14) and questions on degree of satisfaction with the allocated app.

### Intervention and Control Apps

#### TeleCoach

**Figure 2:**
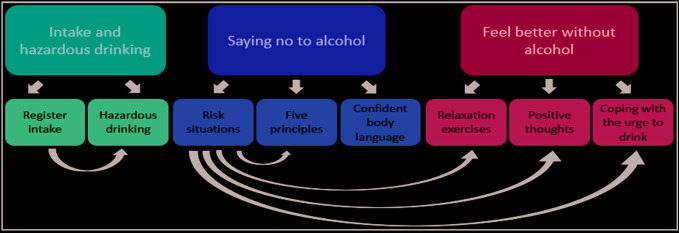
TeleCoach app structure with component connetions.

The intervention app, TeleCoach, is a smartphone app consisting of three major components, concerning self-monitoring, relapse prevention and emotion regulation. **Figure 2** shows the structure of the app with its main components and their subcomponents. Following login, users see a menu of the three main components on the home screen. Selecting any component leads to a new list of subcomponents. The three main components can be reached independently, and some sub-components are linked to each other depending on what the user has registered in the previous component.

The first component, titled “Intake and hazardous drinking”, consists of two components. The first is “Register intake”, consisting of a Timeline followback registration form where users report their daily drinking for the past 7 days. The second is “Hazardous drinking”, where users who register excessive drinking at more than nine (women) or fourteen (men) standard drinks in the past week receive information regarding hazardous drinking and its consequences. This information is followed by a short form containing questions regarding how users perceive their own alcohol consumption as well as how motivated they feel about reducing their alcohol intake. Self-monitoring is the most commonly used component in mobile phone apps for health interventions (51). According to Albert Bandura's Social Cognitive Theory, the first step towards a behavioral change is monitoring one's pattern of behavior (52).

The second component of TeleCoach is titled “Saying no to alcohol”. It consists of three parts where the first, “Risk situations”, aims at identifying risk situations. Twelve questions from the AASE are answered to identify the users' potential risk situations for alcohol consumption. Based on these answers, the user is presented with proposed exercises to cope with the situations where they feel the least capable of abstaining from drinking. The second part, “Five principles” (53) offers the user five different ways of declining when offered alcohol, followed by questions concerning the user's self-efficacy for saying no. The third part, “Confident body language”, provides the user with information on how to be perceived as more confident when saying no. The “Saying no to alcohol” component derives from the relapse prevention (RP) model originally developed 35 years ago by Marlatt and George (54). RP is a framework of cognitive-behavioral parts designed to facilitate behavioral change through guidance in how to handle setbacks during the process (55). RP has been evaluated through meta-analyses and shown positive effects, especially when used for alcohol-related problems. RP is a common component of conventional face-to-face interventions regarding substance use (56).

The third and final component of TeleCoach contains exercises under the title “Feel better without alcohol”. This component includes three parts: “Relaxation exercises”, used with the aim of teaching the user how to relax without consuming alcohol, “Positive thoughts”, a training task for eliciting positive thoughts for users who often have negative thoughts about themselves, and “Coping with the urge to drink”, an urge surfing technique. These three strategies focus mainly on enhancing the user's emotion-regulation skills and introduce new ways of coping with distress and cravings, replacing the use of alcohol as a stress-reducing and positive emotion enhancing tool. The relaxation exercises and urge surfing technique taught in the app are based on the theory of mindfulness, an area that has seen a dramatic increase of clinical research in the past two decades and shown positive effects on several health outcomes (57). A growing number of studies have examined the effects of mindfulness techniques on reducing stress levels and reducing cravings in recent years (58). These studies have shown promising results for the effects of both long-term and brief mindfulness interventions in treating substance misuse and enhancing relapse prevention (59, 60). “Positive thoughts”, the third exercise in the “Feel better without alcohol” component, targets users who often experience having negative thoughts about themselves. This negative mindset can cause users to drink in order to cope with the negative emotions. By having the user focus on positive aspects and occurrences in their daily life the exercise aims to shift focus to a more positive mindset and evoke positive thinking. The positive thoughts intervention is based on the research field of positive psychology which focuses on how positive emotions, positive character traits and enabling institutions contribute to individuals' well-being (61). Using interventions in which participants are asked to think about and write down things that go well for each day has shown positive results in increasing happiness and lowering depression (62). See **Figure S1** for screenshots.

#### Control

The control app contains information on hazardous drinking and its consequences. The information includes early telltale signs of risk consumption as well as signs of severe risk consumption. The control app differs from the intervention app in that it does not offer the user any active components or skills training options. The information in the control app derives from the alcohol-related component in a lifestyle-improvement method evaluated in a large observational study in primary care and was used by permission (63). See **Figure S2** for screenshots.

### Ethical Considerations

All participants received detailed written information about the study and gave their informed consent to participate. They were also informed that they could cancel their participation at any time without explanation. Personal data such as email address, gender and age were not stored in connection with any outcome data. All persons involved in the research project were covered by professional confidentiality. Ethical approval for the study was given by the Swedish Ethical Review Authority (approval number 2016/1088-31, amendment number 2018/2569-32).

### Statistical Analyses

IBM SPSS Statistics version 25 for PC was used for all statistical analyses (64). Descriptive statistics were used to present group differences between the control and treatment group for gender, age, marital status, education, occupation, duration of alcohol problems, and previous help-seeking. For comparison of alcohol consumption measures, descriptive statistics were presented of differences in number of drinks past week, standard glasses consumed per typical week, drinking occasions per typical week, number of binge occasions per typical week, average eBAC per typical week, peak eBAC within the past month and perceived alcohol abstinence self-efficacy. Continuous variables were compared using independent samples t-tests and categorical variables were compared using chi-square tests.

Repeated measures analysis of variance (ANOVA) was used to measure changes in primary and secondary outcome measures from baseline to follow up and to examine any Group x Time interaction effects. Cohen's *d* effect sizes were calculated using an effect size calculator (65). App usage in the intervention and control participants was compared using an independent samples t-test on total number of visits, number of weeks between first and last visit, mean time per visit and total time spent using the app. Further analyses compared use of the eight TeleCoach components in the intervention group, yielding descriptive statistics regarding number of visits, mean time spent in app, total time spent in app and number of visits in each of the eight components. All app usage analyses concerned participants who had access to the app for at least a month. At the time of app usage data extraction, one participant had only had access to the app for 1 week and was excluded from these analyses.

A sensitivity analysis was performed comparing the initial screening assessment between participants who were distributed the baseline assessment survey and completed it, and those who did not complete it. The analysis showed no difference between the two groups in participant characteristics, alcohol consumption in the past week, symptoms of depression and drug usage. A sensitivity analysis was also performed for participants who completed the follow-up assessment and those who did not respond. No difference was shown in baseline participant characteristics.

## Results

### Participant Characteristics

Participant demographic characteristics from the screening and baseline surveys are shown in **Table 1**. Participants' mean age was approximately 49 years (*M* = 48.93, *SD* = 11.88). More than two thirds of the participants were women (69.7%). A majority of the participants had a high educational level (undergraduate studies or higher, 51.7%) whereas only 6.7% of the participants had an educational level of completing junior high school. Among the 34 participants (39.3%) who had previously sought help concerning their problematic alcohol consumption, 10 had received medication such as Campral, four had attended Alcoholics Anonymous meetings and six had received psychotherapy. Another 14 participants reported seeking other forms of help, for example from their municipality or alcohol clinics.

**Table 1:** Participant demographic characteristics at screening and baseline assessment.

Characteristic	Controls (n=47)	Treatment (n=42)	Total (n=89)	*p* values*
Women (%)	70.20	69.00	69.70	.91
Age: *M* (*SD*)	49.43 (11.20)	48.38 (12.70)	48.93 (11.88)	.68
Marital status (%)				.95
Married	68.10	69.00	68.50	
Widowed	12.80	11.90	12.40	
Single	19.10	19.00	19.10	
Education (%)				.66
Junior high school^a^	6.40	7.10	6.70	
High school^a^	34.00	45.20	39.30	
Undergraduate^a^	51.10	33.30	42.70	
Graduate^a^	6.40	11.90	9.00	
Other	2.10	2.40	2.20	
Occupation (%)				.08
Working	93.60	73.80	84.30	
Sick leave	0.00	7.10	3.40	
Seeking	2.10	4.80	3.40	
Retired	4.30	9.50	6.70	
Parental leave	0.00	2.40	1.10	
Other	0.00	2.40	1.10	
Duration of alcohol problems (%)				.69
0–1 year	2.10	4.90	3.40	
1–2 years	27.70	26.80	27.30	
3–5 years	34.00	43.90	38.60	
6–10 years	17.00	12.20	14.80	
More than 10 years	19.10	12.20	15.90	
Help before (%)				.90
Yes	40.40	38.00	39.30	
No	59.60	61.00	60.20	

a Junior high school, Primary secondary education (mandatory); High school, Upper secondary education; Undergraduate, Bachelor's level education; Graduate, Master's level education or higher. *P-values for between-group comparisons based on t-tests.

Participants' clinical characteristics are shown in **Table 2**. Following study inclusion, participants in the intervention and control groups did not differ significantly in alcohol use disorder criteria, symptoms of hazardous drinking, alcohol cravings, or comorbidity in the form of problematic drug use, depression or anxiety. However, a significant difference did occur in participants' readiness to change, with the intervention group scoring significantly higher with a mean of 9.05 (*SD* = 1.61) in comparison to the control group, which scored 8.13 (*SD* = 2.32) with a small between-group effect size (*t*(86) = 2.14, *p* =.04, Cohen's *d* =.46).

**Table 2:** Recruited participants' clinical characteristics at screening and baseline.

	Intervention		Control		Total	
Parameter (score range)	*N*	*M* (*SD*)	*N*	*M* (*SD*)	*N*	*M* (*SD*)
AUD (0–11)	42	6.43 (2.37)	46	6.20 (2.37)	88	6.31 (2.36)
AUDIT (0–40)	42	18.36 (6.70)	46	18.28 (5.24)	88	18.32 (5.94)
PACS (0–30)	42	14.40 (6.24)	46	14.24 (5.92)	88	14.32 (6.04)
DUDIT (0–44)	42	0.50 (2.00)	46	0.28 (0.91)	88	0.39 (1.52)
MADRS-S (0–54)	42	16.71 (8.43)	46	15.91 (8.56)	88	16.30 (8.46)
GAD-7 (0–21)	42	6.83 (4.38)	46	5.48 (4.29)	88	6.13 (4.36)
Readiness ruler(0-10)	42	9.05 (1.61)	46	8.13 (2.32)	88	8.57 (2.05)

### Outcomes

#### Attrition

At baseline assessment, three participants had partially missing data. One participant had not filled in the DDQ at all and another had filled in the peak consumption for DDQ but not the values per day for a typical week. One final participant reported extreme values for DDQ per day but had valid data values for peak consumption for DDQ. The missing data and the extreme values were imputed using the mean values for the participant's allocated group.

At 6-week follow-up, four participants did not complete the entire 6-week follow-up survey and had valid data only for the Alcohol consumption measure of TLFB. The four participants' data were included for the analysis of variance and calculation of effect sizes for alcohol consumption. Missing data for the remaining outcome measures were not imputed.

#### Primary Outcome

There were no significant group differences in the primary outcome measure of alcohol consumption, measured with TLFB, at baseline or 6-week follow-up. Significant within-group decreases over time from baseline to 6-week follow-up were shown for both intervention and control groups [*F*(1, 55) = 43.98, *p* < .001]. However, the between-group Time x Group interaction effect was non-significant on alcohol consumption [*F*(1, 55) = 3.20, *p* =.08]. Further analyses of within-group effect sizes for alcohol consumption by time showed large effects for both the intervention (Cohen's *d* = 1.37) and control (Cohen's *d* = 0.92) groups. See **Table 3**.

**Table 3:** Baseline and follow-up primary and secondary outcome measures of alcohol consumption and abstinence self-efficacy.

Parameter	Baseline						6-week follow-up						Baseline - six-week follow-up			
	Intervention		Control		Total		Intervention		Control		Total		Within-group analysis^a^		Intervention	Control
	N	M (SD)	N	M (SD)	N	M (SD)	N	M (SD)	N	M (SD)	N	M (SD)	F (df)	*P*	Cohen's d^b^	Cohen's d^b^
Alcohol consumption	42	32.73 (21.16)	47	26.00 (14.08)	89	29.17 (17.99)	26	12.73 (10.52)	31	13.48 (11.13)	57	13.14 (10.77)	43.98 (1, 55)	<0.001	1.37	0.92
Quantity	42	26.00 (19.55)	47	22.99 (15.33)	89	24.41 (17.41)	22	11.68 (11.83)	31	13.60 (9.93)	53	12.80 (10.69)	23.01 (1, 51)	<0.001	0.87	0.71
Frequency	42	4.64 (1.68)	47	4.58 (1.69)	89	4.61 (1.67)	22	2.73 (2.31)	31	3.10 (1.85)	53	2.94 (2.04)	42.80 (1, 51)	<0.001	0.91	0.85
Binge occasions	42	3.05 (2.05)	47	2.96 (1.96)	89	3.00 (1.99)	22	1.59 (2.15)	31	1.81 (1.72)	53	1.72 (1.90)	19.39 (1, 51)	<0.001	0.72	0.57
Average eBAC	42	0.75 (0.44)	47	0.75 (0.49)	89	0.75 (0.46)	22	0.48 (0.52)	31	0.47 (0.43)	53	0.47 (0.46)	15.43 (1, 51)	<0.001	0.66	0.39
Peak eBAC	42	1.42 (0.67)	47	1.50 (0.80)	89	1.46 (0.73)	22	0.88 (0.72)	31	1.05 (0.82)	53	0.98 (0.78)	15.74 (1, 51)	<0.001	0.98	0.47
AASE total	42	2.82 (0.64)	47	2.53 (0.84)	89	2.66 (0.76)	22	2.68 (0.63)	31	2.98 (0.82)	53	2.86 (0.76)	0.88 (1, 51)	.35	0.10	0.32
AASE NA^c^	42	2.45 (0.91)	47	2.33 (1.03)	89	2.39 (0.97)	22	2.60 (1.09)	31	2.89 (1.12)	53	2.77 (1.11)	4.59 (1, 51)	.04	0.18	0.37
AASE PO^c^	42	3.24 (1.15)	47	2.91 (1.15)	89	3.07 (1.15)	22	3.15 (0.90)	31	3.22 (0.97)	53	3.19 (0.93)	0.20 (1, 51)	.65	0.04	0.08
AASE SP^c^	42	2.50 (1.00)	47	2.13 (0.98)	89	2.31 (1.00)	22	2.24 (0.90)	31	2.66 (0.93)	53	2.48 (0.93)	0.76 (1, 51)	.37	0.14	0.38
AASE WU^c^	42	3.07 (0.79)	47	2.74 (0.98)	89	2.90 (0.90)	22	2.73 (0.90)	31	3.16 (0.98)	53	2.98 (0.96)	0.22 (1, 51)	.64	0.38	0.22

^a^Results from Repeated measures ANOVA. ^b^Within-group effect size over time. ^c^NA, Negative affect; PO, Physical & other concerns; SP, Social positive; WU, Withdrawal and urges.

#### Secondary Outcomes

##### Alcohol Consumption

The five secondary outcome measures of alcohol consumption show no significant between-group differences at baseline or 6-week follow-up, but all secondary outcome measures showed significant within-group decreases over time for both intervention and controls. Measures of within-group effect sizes showed large effects in three of the five alcohol consumption measures for the intervention group: Quantity (Cohen's *d* = 0.87), Frequency (Cohen's *d* = 0.91) and Peak eBAC (Cohen's *d* = 0.98); and medium effect sizes for the remaining two: Binge occasions (Cohen's *d* = 0.72) and Average eBAC (Cohen's *d* = 0.66). In the control group, one out of the five measures showed large effects: Frequency (Cohen's *d* = 0.85); two showed medium effect on Quantity (Cohen's *d* = 0.71) and Binge occasions (Cohen's *d* = 0.57); and the remaining two showed small effects: Average eBAC (Cohen's *d* = 0.39) and Peak eBAC (Cohen's *d* = 0.47). Nominal differences between within-group effect sizes favored the intervention group for these outcomes.

The measure of self-efficacy in abstaining from drinking showed non-significant change over time for the total score of AASE, [*F*(1, 51) = 0.88, *p* =.35]. However, out of the four subscales, a significant increase over time was seen in both groups for self-efficacy in regard to negative affect, [*F*(1, 51) = 4.59, *p* =.04]. Nominal differences between within-group effect sizes favored the control group, except for self-efficacy in regard to withdrawal urges, where the difference favored the intervention group.

##### App Usage

Analyses of app usage showed no significant differences between the two groups for users with access to their respective app for at least 1 month. On average, participants in the intervention group used the app for no more than 2 weeks. **Table 4** shows data on app usage, comparing the two groups.

**Table 4:** App usage data with group differences calculated via independent samples t-test.

Parameter	Intervention (n=41) M (SD)	Control (n=44) M (SD)	Total (n=85) M (SD)	*df*	*t*	*p*	Cohen's d*
**Total number of visits**	3.56 (4.13)	4.39 (4.89)	3.99 (4.53)	83	-0.84	.40	0.18
**Number of weeks from first to last visit**	1.15 (2.31)	2.05 (2.58)	1.61 (2.48)	83	-1.69	.10	0.37
**Mean time per visit (HH : MM:SS)**	0:06:21 (0:05:52)	0:06:36 (0:08:59)	0:06:28 (0:07:36)	83	-0.16	.88	0.04
**Total time** **(HH : MM:SS)**	0:20:36 (0:30:17)	0:31:24 (1:03:57)	0:26:12 (0:50:35)	83	-0.98	.33	0.22

*Between-groups comparison.

Further analyses of app usage in the intervention group showed that out of the eight components, the “Register intake” component, in which participants complete a TLFB form, was the one most commonly used. Out of the active app components, where participants engage in different forms of exercises aimed at lowering alcohol consumption, “urge surfing” in the “Coping with the urge to drink” component was the most used. **Table 5** provides a summary of number of users and mean number of visits per component.

**Table 5:** Number of participants accessing components and number of visits for the intervention app (see for an overview of the app).

Intervention app component	*N* (%)	Mean number of visits	*SD*
Register intake	32 (78)	1.94	1.70
Hazardous drinking	30 (73)	2.00	1.44
Risk situations	27 (66)	1.74	1.13
Coping with the urge to drink	27 (66)	1.59	1.05
Positive thoughts	25 (61)	1.56	1.16
Relaxation exercises	23 (56)	1.57	0.95
Five principles	22 (54)	1.27	0.55
Confident body language	17 (42)	1.29	0.69

## Discussion

Controlled trials on smartphone app studies for adult internet help-seekers with problematic alcohol use are scarce and support for app effectiveness is unclear. The overall purpose of this pilot trial was to prepare for a full randomized controlled trial by assessing possible effects on the primary outcome of number of drinks in the past week; secondary aims concerned additional alcohol outcomes, the level of app usage in intervention and control groups, and the level of participant comorbidity and establishment of routines for its management. The findings show that alcohol consumption declined with large effect sizes in both intervention and control groups, nominally favoring the intervention group but lacking between-group statistical significance. Secondary outcomes showed the same pattern, but with small to medium effect sizes. Interestingly, self-efficacy increased in relation to negative alcohol effects in both groups, nominally favoring the control group, with a small effect size. App usage data showed that both the intervention and control apps were used approximately equally, for up to 2 weeks, with an average total of four visits to the app and approximately 6 min spent per visit and a total time spent of less than 30 min. Co-morbidity levels at baseline were low regarding drug use, but the clinical severity levels of depression and anxiety were moderate. Motivation to change was very high in both groups, and significantly higher for the intervention group in comparison to the control group.

In terms of continuation with the planned large RCT, the nominally differing within-group effect sizes found in the primary outcome suggest that a larger study could be worthwhile to complete. A power analysis based on the pilot findings showed that with a significance level of α=0.05 and 80% power, a total of at least 100 participants per group will be needed at 26-week follow-up in order for the current non-significant between-group effect size of *d*=0.24 to be significant (see **Figure S3**). The planned baseline recruitment for the RCT has been pegged at up to 1,000 participants in total and taking attrition into account we expect to satisfy power requirements for identifying any significant between-groups effect. Even if the small and non-significant between-groups effect size identified in the pilot trial persists in the RCT, the power calculation suggests significance will be achieved, corresponding to recent results from a individual patient data meta-analysis showing an overall between-groups effect size of *g=*0.26 for internet-based interventions for adult problem drinking in comparison to control groups (10). An addition positive outcome of the pilot trial is the revision of inclusion criteria to include potential participants with risky alcohol use criteria according to the AUDIT and no upper limit for AUD criteria. This strategy attracted a sample with a mean AUDIT score of just over 18, indicating that participants had harmful alcohol use rather than the more severe probable dependence found in samples with more stringent inclusion criteria, in trials offering an internet intervention of at least 10 weeks [e.g., (15)].

Regarding the issues of app usage and engagement, over the 6-week pilot trial period both the intervention and control groups engaged with the app on an average of four occasions, for about 6 min each time, over a period of about 2 weeks, with an average of 26 min spent actually using the app and, surprisingly, a nominal, non-significant “advantage” for the control app. The latter app contained minimal information on reducing hazardous or harmful alcohol consumption, previously used in self-help material offered in primary care together with a behavior change counseling session (63). We would have expected intervention app users, who had access to several interactive tasks, to spend more time practicing their tasks on the app than control app users, who could basically read information and indicate their preferred behavior change tips by checking a box. Although all users had access to their respective app for at least 1 month, the mean number of weeks they actually used the app were less than two, i.e., less than half of the period available. To put these numbers in perspective, reports on smartphone usage in the UK and U.S. show that adults spend an average of 2 h and 28 min and 2 h and 22 min, respectively, per day on their smartphones (66, 67). Nonetheless, a systematic review of self-help digital devices for management of long-term conditions of ill-health showed that the time spent using app features was not related to outcome (68). The planned RCT should indicate whether the possibly greater amount of time spent on the control app persists, and may shed light on any possible advantages of the control app, which is based on informational material associated with positive health outcomes among primary care patients (63).

Comorbity is an additional factor that needs to be taken account in relation to outcomes. However, in this pilot trial our focus regarding comorbidity concerned feasibility of the screening and recruitment methods in preparation for the planned large RCT, rather than associations between comorbidity and outcomes. About one-third of the 16 participants who met comorbidity exclusion criteria responded to contact attempts and were included in the study after a telephone interview. The interview procedure was developed to begin with an explanation of the study rationale, feedback on the immediate reason for the interview in terms of particular exclusion critieria, followed by an invitation for the potential participant to describe their current situation and any existing treatment provider contacts for the depression, suicidality, or drug use identified. A brief MI-based intervention followed, where the respondent was asked how confident they were regarding change in their alcohol consumption and whether they had taken any steps to make changes, followed by a motivational summary of the next steps for reducing problematic alcohol use and/or addressing mental health or drug treatment needs. At the end of the interview, the function of potential study participation in relation to the participant's situation was discussed, information about coming telephone interviews at 6-, 12-, and 26-week follow-ups was given, and information about the National Alcohol Helpline was also given in case the user felt a need for more in-depth MI-based assistance. All telephone interviews in the pilot trial led to participant inclusion. Participants included despite comorbidity were retained for analysis in the results of this pilot trial cohort but will be analyzed separately in the large planned RCT, for possible inclusion in the main cohort but also to assess special needs, app usage and outcomes in this group. This analysis will have an exploratory character, as comorbidity may be associated with difficulty in changing behavior, but the motivational support offered in this study to participants with comorbidity may facilitate change in comparison to participants without comorbidity who did not receive such support.

### Strengths and Limitations

This pilot trial had several strengths, including low threshold access for internet help-seekers with at least hazardous alcohol use, measurement of app usage, and attention to comorbidity and development of a procedure to address it within the trial. Given that 60% of the participants in the trial reported not having sought help earlier to reduce their alcohol consumption, it could be that this type of highly motivated individuals could easily be reached by the app-based intervention, eventually facilitating treatment-seeking behavior if needed following app access, and thus potentially narrowing the treatment gap for alcohol problems. Low-threshold interventions of this sort and the anonymity offered to users are two factors that may help reduce these barriers.

One possible limitation is that the study attracted participants with harmful alcohol use, on average, but also included participants with hazardous use as well as probable dependence. Problem severity could affect outcome, a possibility we did not examine at this pilot stage but will address in the larger planned RCT. Earlier research has shown, however, that even minimal interventions can be associated with beneficial outcomes for participants at hazardous and harmful levels of use, as well as probable dependence (14). Also, some technical limitations were identified at the pilot trial stage. One technical limitation was that two participants reported difficulties in accessing the app, a factor that might be related to the web-based format of the app, which meant that it had to be saved to the user's home screen *via* the web browser, not downloaded from App Store or Google Play. This limitation was addressed by further clarification at the end of the baseline assessment survey at which point randomization to the app takes place; a general review of the instructions provided to participants has been conducted for the large RCT. A second, app-related limitation was that logon codes that were not used within 30 min of distribution were reset. Eight participants were not able to access the app they had been assigned either because they entered/saved the wrong code at the end of the baseline assessment form or not did not log in to the app in time. The time limit of the codes was not originally stated anywhere in the baseline assessment survey, but was added during the pilot study in preparation for the larger RCT. Due to regulations on data protection and privacy, participants could not use their email address as usernames for their login as this would have led to sensitive information being accessed by the app developers. Given that a randomized code must be assigned and saved by the participants, attrition due to human error is potentially higher than in trials where the trial apps are available on the App Store/Google Play (e.g., 24). A possible solution to this issue would be to create an automated email which sends the code to the user; however due to limitations in the trial survey system this was not possible to remedy

## Conclusions

In conclusion, the present study shows promising results in terms of the need for continued data collection in the larger, randomized controlled trial, which in effect is a continuation of the randomized pilot study reported herein. Clearly, adult internet help-seekers are attracted by the prospect of using an app-based intervention targeting hazardous alcohol consumption. In the pilot design as well as the planned larger trial, the target sample consists of anonymous, highly motivated help-seekers. Previous studies have shown that high scores for readiness to change are associated with improved alcohol consumption patterns (45, 69). Prior research on smartphone apps targeting university students has shown that motivation is a key factor associated with positive outcomes (22); for less motivated participants Motivational Interviewing (28) has shown positive effects for university students in combination with feedback on their drinking levels (70). The reduced alcohol consumption over time noted within groups in this pilot trial is most probably mediated by participant motivation and readiness to change in addition to engagement with the respective apps, particularly in view of the restriction of the current analysis to completers of the 6-week follow-up. The interconnections between engagement, motivation, app usage, and outcomes according to an Intention-to-Treat (ITT) approach remain to be further elucidated, and the planned large RCT should contribute valuable data in this regard.

## Data Availability Statement

The raw data supporting the conclusions of this article will be made available by the authors to any qualified researcher.

## Ethics Statement

The studies involving human participants were reviewed and approved by Swedish Ethical Review Authority (approval number 2016/1088-31, amendment number 2018/2569-32). The patients/participants provided their written informed consent to participate in this study.

## Author Contributions

Authors AB, KS, MG, and CA conceived the study design, and MT and PT carried out the analyses within their thesis work for the BSc degree in Clinical Psychology, under AB and OM's supervision as main and co-supervisors, respectively. This manuscript, based on the thesis originally written by MT and PT, was revised and edited by AB with input from CA, OM, MG, MT, PT, and KS. All authors approved the final manuscript and are accountable for its contents.

## Funding

Author AB was funded for salary by Swedish Research Council grant nr K2012-61-P-22131-01-6 and additional project funding came from grant K2012-61X-22132-01-6. The study was further supported by the independent Swedish Alcohol Monopoly Research Council, grant nr 2015-0097 and partly funded author MG early in the study. The funders did not have any influence on the interpretation of the study results or set any constraints on publishing the results. The principal investigator for all grants is AB.

## Conflict of Interest

AB and CA are co-owners of a company, TeleCoach AB, aiming to disseminate digital interventions for problematic behaviors including hazardous and harmful alcohol use. The company is not currently active.

The remaining authors declare that the research was conducted in the absence of any commercial or financial relationships that could be construed as a potential conflict of interest.
