# Supplementary material for: Reducing Risky Alcohol Use via Smartphone App Skills Training Among Adult Internet Help-Seekers: A Randomized Pilot Trial
Source: Front Psychiatry. 2020 May 27;11:434. doi: 10.3389/fpsyt.2020.00434 (PMC7267061; doi:10.3389/fpsyt.2020.00434)
Supplement: Supplementary file 1 [file Image_1.pdf]

TeleCoach™

INTAG OCH RISKBRUK

KUNNA SÄGA NEJ TILL ALKOHOL

MÅ BÄTTRE UTAN ALKOHOL

Appen TeleCoach™ är utvecklad av Karolinska Institutet och används inom ramen för forskning.

Kontaktinformation

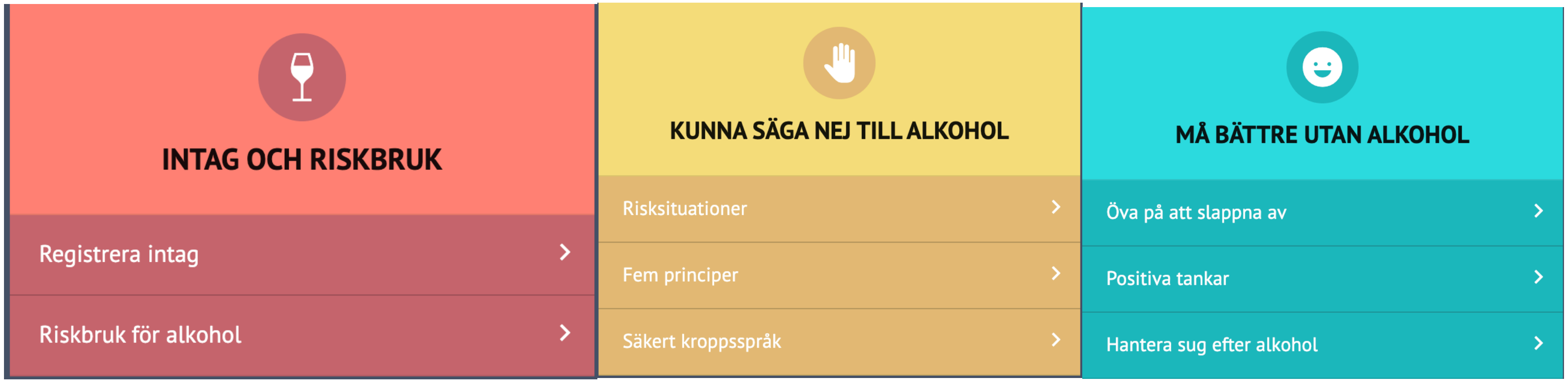

Figure S1. TeleCoach app screenshots, showing the main menu and clickable sub-menus for each section.
