# Supplementary material for: Reducing Risky Alcohol Use via Smartphone App Skills Training Among Adult Internet Help-Seekers: A Randomized Pilot Trial
Source: Front Psychiatry. 2020 May 27;11:434. doi: 10.3389/fpsyt.2020.00434 (PMC7267061; doi:10.3389/fpsyt.2020.00434)
Supplement: Supplementary file 2 [file Image_2.pdf]

# TeleCoach™

## NÄR BLIR DET FARLIGT MED ALKOHOL?

## HUR VET JAG OM JAG HAR ETT RISKBRUK AV ALKOHOL?

Appen TeleCoach™ är utvecklad av Karolinska Institutet och används inom ramen för forskning.

**Kontaktinformation**

## När blir det farligt?

Det finns givetvis gränser för hur mycket människokroppen tål, dessa gränser kan variera mycket från individ till individ.

### Totalkonsumtion

Men om vi utgår från totalkonsumtionen, dvs den sammanlagda konsumtionen, alltså även räknar in en lättöl till maten, finns det vissa gränser man är överens om.

### Berusningsdrickande

Berusningsdrickande bedöms alltid som ”inte helt bra” eller ”risk”.

## Välj kön och läs mer om riskbruk

♂ MAN

♀ KVINNA

Gränserna för alkohol är olika för man och kvinna baserat på den forskning som gjorts. Alkoholgränser finns för ”man” eller ”kvinna”. Därför behöver du

## Tidiga tecken på riskbruk

- Att börja fundera på att ta en eller flera vita veckor, kan för en del vara tecken på att den totala konsumtionen bör ses över.
- Att prata om att sluta helt, men inte just nu, utan längre fram när det passar bättre med humör, stämningar, tid och annat.
- Att börja dricka ensam
- Att ha ett lättväckt alkoholbegär. Dricka lite, utvecklas till mycket. Den första drinken sätter igång beteendet!
- Att för sig själv inte erkänna hur mycket man dricker.
- Att dölja hur mycket man dricker genom tex ta lite större groggar eller flera groggar än andra.
- Att plötsligt märka att alkoholen går svårt åt ekonomin.
- Att dricka före en fest främst för att vara riktigt säker på att få tillräckligt för att bli berusad.
- Att hitta ursäkter för att få anledning att dricka

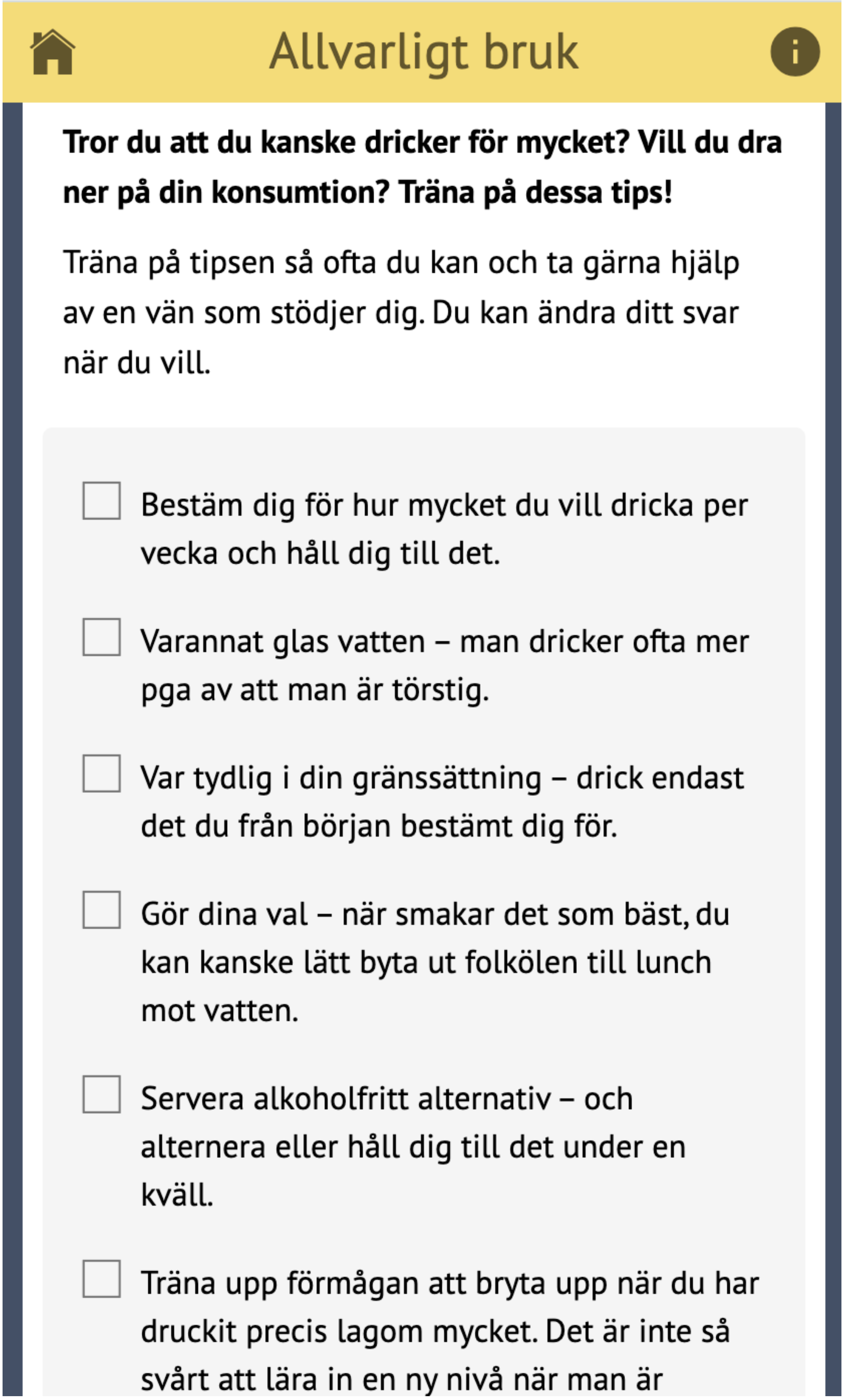


Figure S2. Control app screenshots, showing the main menu and information on when drinking is dangerous, early signs of hazardous use, and tips on reducing consumption, where boxes can be checked to indicate tips suitable for the user
