# Supplementary material for: Reducing Risky Alcohol Use via Smartphone App Skills Training Among Adult Internet Help-Seekers: A Randomized Pilot Trial
Source: Front Psychiatry. 2020 May 27;11:434. doi: 10.3389/fpsyt.2020.00434 (PMC7267061; doi:10.3389/fpsyt.2020.00434)
Supplement: Supplementary file 3 [file Image_3.pdf]

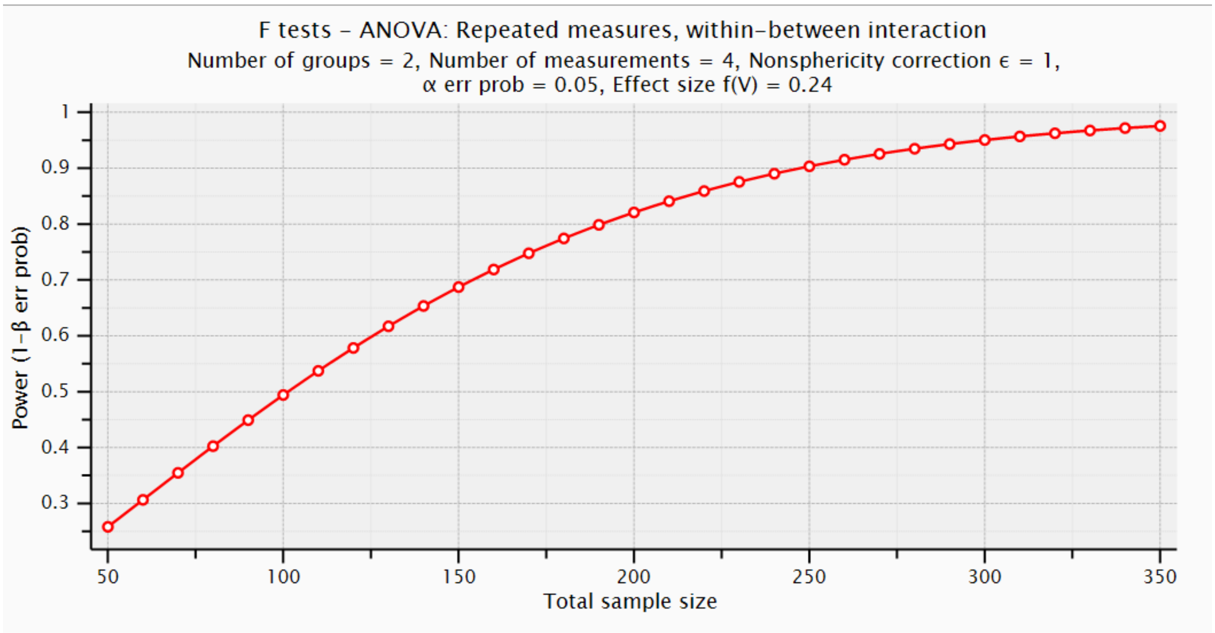

Figure S3. Power calculation based on pilot trial data, extrapolating total needed sample size for planned RCT.
